# Dynamics of stand density and self-thinning in Chinese fir plantations: theoretical insights and empirical validation

**DOI:** 10.3389/fpls.2024.1444807

**Published:** 2024-11-15

**Authors:** Shisheng Long, Siqi Zeng, Guangxing Wang

**Affiliations:** ^1^ Faculty of Forestry, Central South University of Forestry and Technology, Changsha, Hunan, China; ^2^ Department of Geography and Environmental Resources, Southern Illinois University, Carbondale, IL, United States

**Keywords:** self-thinning rule, stand density, tree height, stand density index, Chinese fir plantation

## Abstract

**Introduction:**

Stand density management is essential for adaptive silviculture, thinning decisions, growth modeling, and yield prediction in forestry, particularly for plantations. Despite extensive research on self-thinning rules and the maximum size-density law, significant gaps remain in the biophysical understanding and validation of the relationships among key stand variables and parameters.

**Methods:**

This study theoretically explored and validated the relationship between maximum size-density and two key metrics: average diameter at breast height (D) and tree height (H). We used time-series data from a 30-year clear-cut, fully stocked Chinese fir plantation, a fast-growing commercial species in China, for validation.

**Results:**

A growth balance status for fully stocked stands was proposed, wherein prior to self-thinning, the growth rate of the stand basal area (G) aligns with that of the average tree height (H), expressed as $G'/\left(G-b_{0}\right)=H'/H$ and approaching a constant slope, b_1_. Generalized maximum size-density and stand density index (SDI) equations were developed: $N_{1.0}=A\times D^{-2}$ and $SDI=A\cdot D^{-2}$ with $A=4\times \left(b_{0}+b_{1}H\right)/\pi$ , differing from traditional equations. Additionally, a generalized self-thinning equation, $v=kH^{q}N_{1.0}^{-1}$ or $w=c_{1}H^{q}N_{1.0}^{-1}$ , was introduced, indicating that in fully stocked stands, tree volume or biomass depends on both tree height and tree count.

**Discussion:**

These findings advance understanding of the maximum size-density law and self-thinning boundary, providing refined tools for managing stand density in Chinese fir plantations.

## 1 Introduction

Stand density accounts for the degree of trees occupying and utilizing space in a forest stand and is a quantitative measure of tree stocking. Given a tree species, stand growth varies depending mainly on tree age, site condition, and stand density. Stand density is controllable by forest management. Stand density is thus considered as the most important parameter of plantation management. By adjusting the number of planted trees per unit area, silviculturists can not only determine tree species of forest generation and increase the growth of tree diameter, but also improve merchantable volume and quality of wood and enhance soil and water conservation (Jack and Long, 1996). Determining an optimal stand density for a given management purpose becomes critical. Stand density management thus provides a useful tool for silviculture, thinning, growth and yield prediction, and harvesting (Burkhart, 2013; Jack and Long, 1996; Zeide, 2005).

Stand density can be expressed using an absolute and relative value. Absolute stand densities include the number of trees, basal area, and stocking per unit area. Relative stand densities consist of stand density index (SDI), degree of tree canopy closing, relative spacing index, and stem stock density. The most widely used stand density measures are the number of trees per unit area, SDI, and degree of tree canopy closing. Since the maximum size-density equation was proposed by Reineke (1933), both the maximum size-density law and SDI have been widely used to account for the natural mortality of trees in a pure stand of crown closure or fully stocked pure stand (Yang and Titus, 2002). Based on the maximum size-density law of Reineke, there is a relationship between number of trees per unit area (N) and quadratic mean diameter at breast height (D): N ∝ D. That is, their log–log transformation is a straight line, implying maximum size-density or a self-thinning boundary line (Reineke, 1933; Vospernik and Sterba, 2015): lnN=k+blnD . It is assumed that the relationship exists with a constant slope, b, of −1.605, independent of tree species, age, location, and site condition, while the intercept parameter k varies with tree species (Burkhart, 2013; Burkhart and Tomé, 2012). The maximum size-density law and SDI equation have been widely applied to developing forest growth models, density management diagrams, and forest management plans (Jack and Long, 1996; de Prado et al., 2020). Despite its utility, substantial debate persists regarding several assumptions of the model.

One key point of contention is the omission of tree height (H) as a critical factor in the maximum size-density relationship. Empirical evidence suggests that while tree D has an inverse relationship with N, tree H is often positively correlated with N (Drew and Flewelling, 1979). This suggests that tree height plays a substantial role in determining stand density, which has not been explicitly addressed in Reineke’s model. Moreover, the SDI equation lacks a biophysical explanation for why the slope parameter (b) should remain constant across different tree species and site conditions. Indeed, studies have demonstrated that the slope value may vary depending on tree species, datasets, and methods used. For example, Vanclay and Sands (2009) derived a slope value of −2 for 29 plots of *Eucalyptus pilularis*, while Pretzsch and Biber (2005) reported different slope values for various species in Germany, ranging from −1.789 for common beech to −1.424 for common oak. Additionally, Yang and Burkhart (2017) found a slope of −1.455 for loblolly pine. Furthermore, studies have indicated that the maximum stand carrying capacity may also vary with site-specific factors such as climate (de Prado et al., 2020; Zhang, 2018).

In response to these limitations, Zeide (1985) proposed a modified SDI equation that accounted for the influence of tree height on stand density. The modified equation assumed that the diameter at breast height (D) was related to the length from the crown base to breast height, with this length being proportional to H. When compared to the original SDI model, the modified model demonstrated improved performance (Zeide, 1995). Inoue and Nishizono (2004) proposed an uneven-aged forest growth model, which revealed the mechanisms behind different slopes in the maximum size-density line for uneven-aged stands. The authors suggested that this model generalized the relationship between N and D, while the original SDI model represented a specific case of this generalized model.

Yoda et al. (1963) proposed the self-thinning rule to describe the relationship between average plant biomass or stem volume and stand density. The self-thinning rule is based on the assumption of geometric similarity, implying a consistent relationship between mean biomass and the approximately −3/2 power of stand density in overcrowded, even-aged stands. This relationship is known as the −3/2 power law and has been regarded as a general growth law for plant ecosystems (White and Harper, 1970; Gorham, 1979; White, 1980; Westoby, 1987). However, some studies have questioned the data and statistical methods used to derive the self-thinning rule (Weller, 1987a; Zeide, 1987; Sackville Hamilton et al., 1995) and raised concerns about the validity of the evidence supporting this model (Mohler et al., 1978; Weller, 1987b). Empirical studies have reported that the self-thinning exponents vary depending on species and site conditions (Osawa, 1995; Kikuzawa, 1999). Zeide (1985) demonstrated that site quality significantly affected the self-thinning component, with better site conditions resulting in steeper slopes. These findings indicate that the commonly assumed universal self-thinning exponent (−3/2−3/2−3/2) is not universally applicable, with studies reporting exponents ranging from −1.54 to −2.33, depending on species and environmental factors. For instance, Fang (1992) found that when the average stand biomass increment reached its maximum, the self-thinning exponent approached −1, suggesting that biomass per unit area becomes constant at this point.

Weiner and Thoms (1986) argued that the self-thinning rule arises from asymmetric competition for light, where taller trees shade smaller individuals, ultimately leading to their mortality when light is insufficient for growth. Such asymmetric competition becomes more pronounced under high stand densities, leading to deviations from the assumptions of geometrical similarity. This underscores the need to revisit the self-thinning rule and develop models that account for the dynamic nature of competition within forest stands.

In addition to using average diameter (D) (Reineke, 1933) and volume (v) (Yoda et al., 1963), the maximum size-density law or self-thinning rule can also be expressed using tree height (Hart, 1926). The Hart index, which represents the ratio of mean distance between trees to stand height, suggests that height plays an important role in density dynamics. Studies by Zeide (1985, 1987, 1991, 1995, 2001, 2002, 2005, 2010) and Burkhart (2013) have indicated that quadratic mean diameter is generally the preferred measure for estimating the number of trees per unit area, with mean stem volume and mean tree height being less preferred but still significant (Wilson, 1979; Lynch et al., 2007). This implies that stand average height also affects maximum size-density relationships to some extent.

Another crucial aspect is selecting fully stocked stands for developing maximum size-density or self-thinning boundary lines. Various methods have been proposed, such as visual inspection (Yoda et al., 1963), analysis of mortality over time (Fang, 1992; Weller, 1987a), and relative stand density methods (Solomon and Zhang, 2002). However, these methods often carry uncertainties, and there remains a need for objective, reliable criteria for quantifying the status of fully stocked stands (Bi et al., 2000).

In light of these ongoing debates, the objective of this study is to theoretically justify the relationships between the maximum size-density law and the self-thinning rule by incorporating both average diameter at breast height (D) and tree height (H). This study uses time-series data from a 30-year, clear-cut, fully stocked stand of Chinese fir plantation, one of the most important and fastest-growing commercial tree species in China, to validate these relationships. In addition, generalized maximum size-density and self-thinning equations are proposed, incorporating both D and H to provide a more comprehensive understanding of stand density dynamics. Furthermore, the relationship between the growth of stand basal area (G) and height (H) is explored, and guidelines for selecting fully stocked stands are provided. This approach aims to address the limitations of previous models and contribute to the development of more accurate and practical tools for stand density management in Chinese fir plantations.

## 2 Materials and methods

### 2.1 Study area and dataset

The study deals with a Chinese fir (Cunninghamia lanceolata) plantation in Guang-Ping-Xiang of Huitong County, one of the seven provenance seed regions. Huitong County has a humid subtropical monsoon climate with an annual precipitation of approximately 1,400 mm. The topography of the study area is characterized by low mountains and hills with an elevation range of 300 to 500 m and red soils. A square plot of 666.7 m^2^ located in the middle part of a low mountain was selected. The plot had an aspect of northeast and a slope of 30°C with a site index of 16. In the pure stand of Chinese fir, the trees were planted in 1954 and clear-cut in 1984 to study the growth characteristics of the trees. The plant species under canopy included Lithocarpus glaber, Mallotus apelta, and Lindera communis. Stem analysis was conducted using a new method different from the traditional one. Each of the cut trees was vertically split into two parts. Growth rings were determined, their diameters were measured at a 2-m interval along the stem, and heights were recorded at a 2-year period. The volumes of each tree were calculated at a 2-year time interval using a sectional measurement method. Based on the relationship of bark diameter with the diameter inside bark, the values of diameter, basal area, and volume with bark were derived. The values of the basal area and volume for all the trees were summed up to obtain the stand basal area and volume for the periods of every 2 years. The stand average height for every 2 years was calculated using weighting tree heights by basal area.

The number of planted trees in 1954 was 200 for the plot, which is 3,000 per ha. At the age of 10 years, the stand became a canopy closure, that is, fully stocked. Artificial thinning was carried out by removing 23 trees, that is, 11.5% of 200 trees, at 10 and 11 years. The thinning decreased the canopy cover and provided space for the left trees growing. At the time of clear-cutting in 1984, there were 176 remaining trees, with one dead tree discovered among them. Some of the living trees had not yet reached a height of 1.3 m. The dead tree was found to have 22 growth rings, indicating it had died at the age of 22 years. The statistical parameters of the stand are shown in **Table 1**.

**Table 1:** The statistical parameters of the stand trees.

Stand age (year)	Number of trees per ha	Mean D (cm)	Mean H (m)	Basal area (m^2^/ha)	Volume (m³/ha)	Stem form	H × stem form
4	2,625	3.90	2.92	3.1358	35.4225	3.869	12.31
6	2,790	7.03	5.30	10.8294	79.7455	1.389	15.04
8	2,910	9.19	7.44	19.3025	129.9109	0.905	17.47
10	3,000	10.57	9.16	26.3246	181.2946	0.752	19.80
12	2,655	11.51	10.72	27.6252	210.6803	0.711	19.64
14	2,655	12.32	12.08	31.6501	258.6146	0.676	21.40
16	2,655	12.95	13.18	34.9699	299.2383	0.649	22.70
18	2,655	13.44	14.05	37.6663	332.7382	0.629	23.69
20	2,655	13.85	14.69	39.9994	362.7056	0.617	24.68
22	2,640	14.18	15.22	41.6914	387.4261	0.611	25.47
24	2,640	14.47	15.63	43.4141	407.8722	0.601	26.09
26	2,640	14.74	15.97	45.0494	428.6008	0.596	26.85
28	2,640	14.96	16.27	46.4042	445.5517	0.590	27.38
30	2,640	15.18	16.54	47.7790	463.1672	0.586	28.00

### 2.2 Theoretical assumption

For a young stand without closure of tree crowns, trees can freely grow with expansion of their crowns. After stands are fully stocked, trees are limited for expansion of their crowns and start to compete with each other for materials (light, water, and nutrients) (Weller, 1987b). The competition leads to different size trees such as dominant trees, co-dominant trees, average size trees, and suppressed trees. Thus, it can be assumed that for fully stocked stands: (1) there are no crown gaps existing and the stock volume (v) or biomass (w) of individual trees is proportional to the size of tree crown; (2) the volume of tree crown is proportional to its height and projected area of crown; and (3) the increase of stock volume or biomass obeys the self-thinning rule: $v\propto w^{1.0}$ (White, 1981). Because tree diameter at breast height D and height H are easily measured, these two variables together with total volume (V) are critical parameters in forest resource inventory and highly correlated with biomass. Thus, there is $v_{c}\propto H^{q}S$ , $v\propto v_{c}\propto H^{q}S$ (Weller, 1987b) and:


1 $$v=a_{0}H^{q}S$$

where v is the average tree volume, a_0_ and q are the parameters, H is the tree average height, S is the average area of tree crown base, and v_c_ is the tree crown volume. Let N and g be the number of trees per hectare and the average basal area for an existing stand, N_1.0_, G_1.0_, V_1.0_, and P_1.0_ be the number of trees per hectare, the total basal area, the total volume, and stem stock density for a fully stocked stand, respectively. Equation 1


2 $$\begin{array}{c} \text{becomes: }v=a_{0}\times H^{q}\times \frac{10000}{N_{1.0}}\to V_{1.0}=N_{1.0}\times v=10000a_{0}\times H^{q}=k\times H^{q},\text{ and}\\ N_{1.0}=\frac{kH^{q}}{v} \end{array}$$

where $k=10000\times a_{0}$ . Let HF be the tree height timing the stem form. Equation 2 means that $kH^{q}$ is the total volume for the fully stocked stand, $V_{1.0}$ . Thus, there is


3 $$N_{1.0}=\frac{kH^{q}}{v}=\frac{G_{1.0}}{g}\times \frac{HF}{HF}=\frac{G_{1.0}}{g}$$

### 2.3 Theoretical analysis

Basal area-volume tables for fully stocked stands based on the maximum size-density theory have been widely utilized in forest inventory and management to determine stand volume with stem density per unit area in China (Che and Zhang, 2012; Fu et al., 2008; Zhang, 2018). In the tables, it is assumed that for the stands with a stem density of 1.0, that is, fully stocked stands, given a mean height, the total basal area ( $G_{1.0}$ ) and volume are the same regardless of site condition. In particular, in forest management and inventory, the basal area-volume tables can be employed together with angle count sampling to quickly obtain stand stem density, total basal area, and volume. This method is simple and practical. An important model used in the basal area-volume tables is


4 $$G_{1.0}=b_{0}+b_{1}H$$

Based on Equation 4, $G_{1.0}$ is a function of *H*, that is, G=f(H) . Obviously, the *G–H* relationship does not go through the origin of the coordinate system. It can be supposed that the intercept is $b_{0}$ , where $b_{0}$ and $b_{1}$ are the parameters. Therefore, there is:


5 $$N_{1.0}=\frac{G_{1.0}}{g}=\frac{b_{0}+b_{1}H}{g}$$

From $N_{1.0}\times g=G_{1.0}$ , it is known that when the real basal area G=N×g is smaller than or equal to $G_{1.0}$ , and the stock density P is smaller than or equal to $P_{1.0}$ . This indicates that in the stand, there are some gaps that provide space for expansion of tree crowns and the self-thinning does not happen. The trees continue to grow until $G=N\times g=G_{1.0}$ . For plantations, when N×g reaches $G_{1.0}$ , the self-thinning may not happen immediately and there is a certain period of time during which the number of trees does not decrease or increase. For natural uneven-aged forests, the self-thinning may happen immediately, leading to some trees dying and at the same time, new trees may appear due to generation.

Based on Equation 5, when the stand basal area G increases and reaches $G_{1.0}$ , there is


6 $$N_{1.0}=\frac{G_{1.0}}{g}=\frac{b_{0}+b_{1}H}{g}=\frac{b_{0}+b_{1}H}{\frac{\pi }{4}D^{2}}=\frac{4}{\pi }\times \left(b_{0}+b_{1}H\right)D^{-2}$$

When *H* in Equation 6 is fixed, the stand maximum number of trees is


7 $$N_{1.0}=AD^{-2}$$

Equation 7 is similar to $N=kD^{b}$ (Reineke, 1933), but its exponent is −2, instead of −1.605, and $A=\frac{4}{\pi }\times \left(b_{0}+b_{1}H\right)$ , where the parameter A varies depending on tree height *H*.

On the other hand, based on Equation 2: $N_{1.0}=\frac{kH^{q}}{v}$ or $v=\frac{V_{1.0}}{N_{1.0}}$ and $v\propto w^{1.0}$ , there are:


8 $$v=\frac{W}{c_{0}}=\frac{V_{1.0}}{N_{1.0}}=kH^{q}N_{1.0}^{-1},\text{ or }w=\frac{c_{1}H^{q}}{N_{1.0}}=c_{1}H^{q}N_{1.0}^{-1}$$

where $c_{0}$ is the biomass density, k and q are the parameters, $c_{1}=c_{0}\times k$ , and w is the average tree biomass. Equation 8 is similar to the self-thinning rule $v=k\times N^{-\frac{3}{2}}$ (Yoda et al., 1963).

Based on Equation 4, in addition, the basal area per unit tree height $b_{1}=\frac{G-b_{0}}{H}$ . If the $b_{1}$ does not change, there is $b_{1}^{'}={\left(\frac{G-b_{0}}{H}\right)}^{'}=0$ , that is, $b_{1}^{'}=\frac{H{\left(G-b_{0}\right)}^{'}-\left(G-b_{0}\right)H^{\prime }}{H^{2}}=0$ . The following relationship is obtained:


9 $$\frac{G^{\prime }}{G-b_{0}}=\frac{H^{\prime }}{H}$$

where $G^{\prime }$ is the annual increment of the stand basal area, $H^{\prime }$ is the annual increment of the average tree height, and $b_{0}$ is the intercept.

Reference from Equation 9: For a stand with any stand density, as long as its growth of basal area and height meets Equation 9, its relative stand density does not change, which implies that a fully stocked stand can grow in the way of Equation 9 until the relationship of the basal area growth with height growth is broken, that is, self-thinning happens.

## 3 Data validation and results

### 3.1 Relationship of basal area with average height

**Figure 1:**
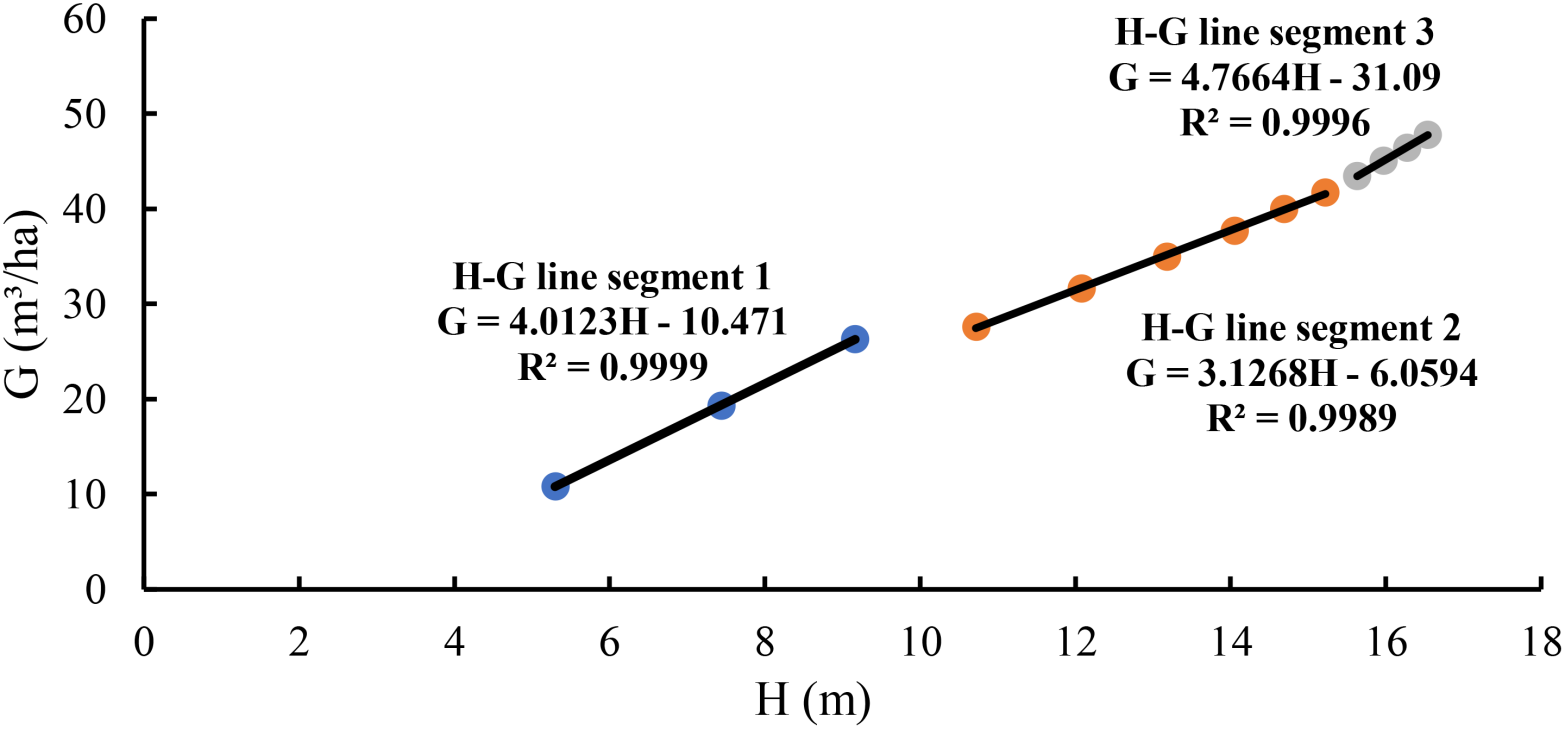
The relationship of the basal area with the average height for the stand.

The scatter plot of stand basal area against average height showed three different relationships with changing slopes (**Figure 1**). Using Equation 4, the relationship of basal area with average height was separately modeled for three line segments. Line segment 1 demonstrated the relationship from 5.3 to 9.2 m of the average height corresponding with ages of 6 to 10 years. At this stage, the tree canopies changed from relative open to closure and natural pruning happened without self-thinning. The artificial thinning that was conducted at the ages of 10 and 11 years released the space for the left trees growing. Line segment 2 had a relatively longer period of time and dealt with the relationship of the basal area with the average height from 10.7 to 15.2 m corresponding to the age from 12 to 22 years. Because the thinning at the ages of 10 and 11 years decreased the stand density and changed the slope of the basal area–average height relationship, it made the slope different at line segment 2 from that at line segment 1. Line segment 3 revealed the relationship of the basal area with the stand average height from 15.6 to 16.5 m at the ages of 24 to 30 years. At this stage, as the trees grew, the tree crowns and diameters got larger, the stand developed towards the fully stocked again, which slowed down the growth of the tree height and increased the slope of the basal area–average height relationship.

### 3.2 Stand maximum size-density line

The above analysis (**Figure 1** and **Table 1**) implied that at the age of 10 years, the canopy cover of the stand became closed and natural pruning started, but self-thinning had not taken place yet. The management thinning halted the self-thinning and provided space for the trees to grow, which led to a longer line segment with an invariable slope of the basal area–average height relationship. There was only one dead tree found at the age of 22 years, indicating that by the year of 22, the stand started to partially get the canopy closure, but not fully closed. By the age of 24 years, the stand average height reached 15.6 m and the stand got fully stocked again, which resulted in the limited space and slowed down the growing of the tree heights.

Therefore, the slope of the basal area–average height relationship from line segment 1 could be regarded as the exponent of the maximum size-density line. When the stand was fully stocked at the age of 10 years and if no thinning was conducted, the trees in the stand would continue to grow for a certain time period and then would start self-thinning. Because of being fully stocked, the trees would grow by changing the ratio of height to *D* and become stable for some years. During this time period, self-thinning did not necessarily occur. However, without the artificial thinning, the tree competition would become stronger and self-thinning would happen when the suppressed trees were not able to obtain enough light, water, and nutrients to grow.

**Figure 2:**
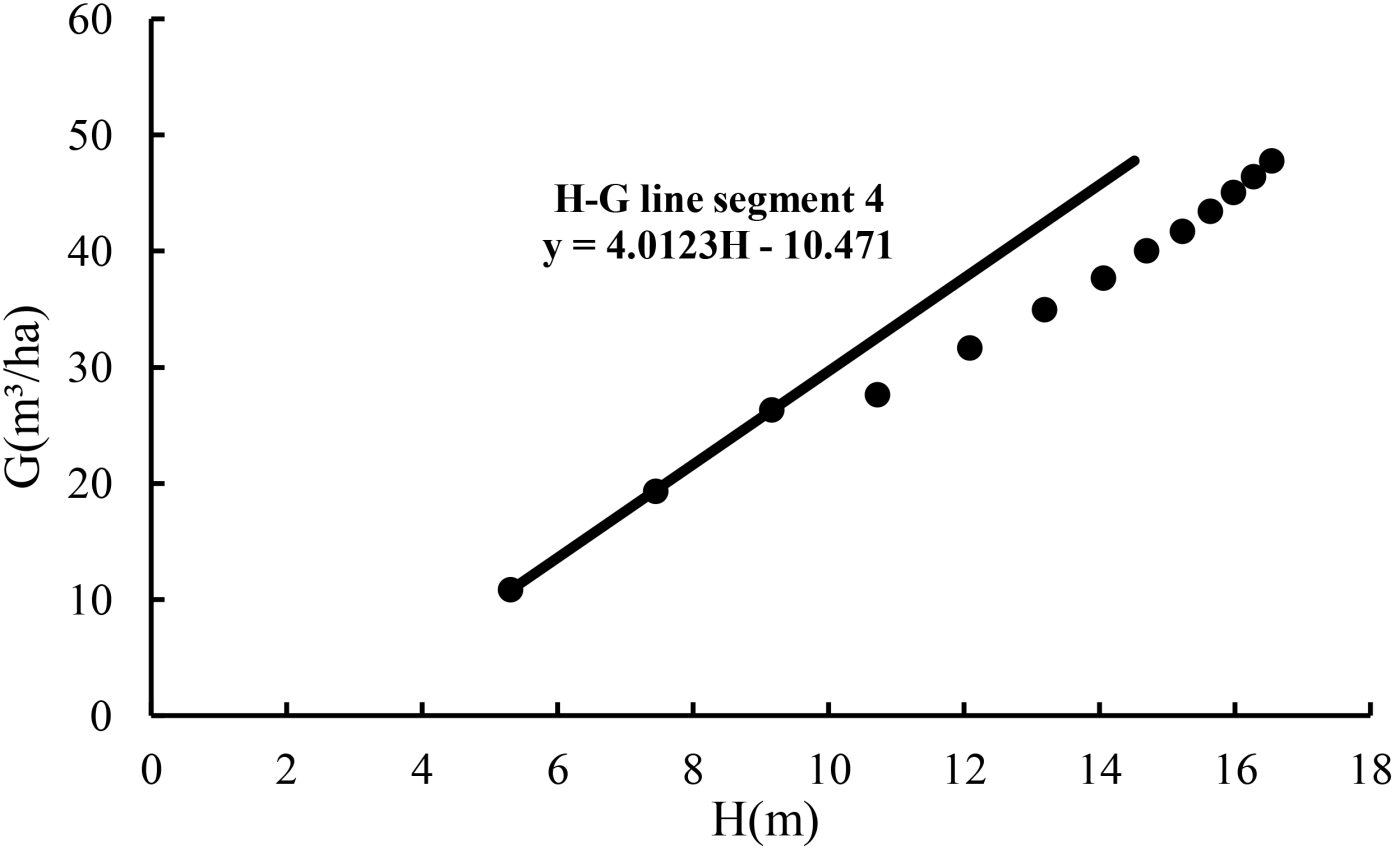
The maximum size-density line based on the relationship of the stand basal area with stand average height for the fully stocked stand.

With the slope of 4.0123, extending line segment 1 resulted in line segment 4 in **Figure 2**, implying the maximum size-density line based on the relationship of the stand basal area with average height for the fully stocked stand. Then, dividing the values of the basal area from line segment 2 and line segment 3 by the values of the basal area from line segment 4 corresponding to the same average heights led to the estimates of stock density *P* with an average estimate of 0.829.

Line segment 4 was used to define a stand growth balance status in which its stock density P_1.0_ = 1.0, the number of trees does not change, and the change rate of the basal area–average height relationship is zero. At the stage of the stand growth balance status, the annual growth rate of the basal area balances the annual growth rate of the average height. That is, $\frac{G^{\prime }}{G-b_{0}}=\frac{H^{\prime }}{H}=b_{1}$ with a change rate of almost zero and meeting Equation 9. Because of the invariable number of trees, this means that the annual growth rate of *D* also balances the annual growth rate of the average height. The results are shown in **Table 2**.

**Table 2:** The changes of the stand parameters over age (Note: D and H are the average diameter at breast height and average height; G' , H' and D' are the annual increment of basal area, average height, and average diameter; H'/H and D'/D are the growth rate of average height and diameter; ΔH is the average increment of average height given a 2-year interval; g is the average tree basal area; g, G' , H' , H/D ,and H'/H were derived based on observations, *G'/(G-b_0_)* was derived based on the line segments).

Age (year)	Trees/ha	D (cm)	H (m)	G’ (cm^2^·yr)	ΔH (m·yr)	H’ (m·yr)	g/H	G’/H’	H/D	G’/(G−b_0_)	H’/H
4	2,625	3.90	2.92	0.784	0.73	0.730	4.09	1.074	0.749	0.058	0.250
6	2,790	7.03	5.30	3.847	0.88	1.190	7.32	3.233	0.754	0.181	0.225
8	2,910	9.19	7.44	4.237	0.93	1.070	8.92	3.959	0.810	0.142	0.144
10	3,000	10.57	9.16	3.511	0.92	0.860	9.58	4.083	0.867	0.095	0.094
12	2,655	11.51	10.72	0.650	0.89	0.780	9.71	0.834	0.931	0.019	0.073
14	2,655	12.32	12.08	2.012	0.86	0.680	9.87	2.960	0.981	0.053	0.056
16	2,655	12.95	13.18	1.660	0.82	0.550	9.99	3.018	1.018	0.040	0.042
18	2,655	13.44	14.05	1.348	0.78	0.435	10.10	3.099	1.045	0.031	0.031
20	2,655	13.85	14.69	1.167	0.73	0.320	10.26	3.646	1.061	0.025	0.022
22	2,640	14.18	15.22	0.846	0.69	0.265	10.38	3.192	1.073	0.018	0.017
24	2,640	14.47	15.63	0.861	0.65	0.205	10.52	4.202	1.080	0.012	0.013
26	2,640	14.74	15.97	0.818	0.61	0.170	10.68	4.810	1.083	0.011	0.011
28	2,640	14.96	16.27	0.677	0.58	0.150	10.80	4.516	1.088	0.009	0.009
30	2,640	15.18	16.54	0.687	0.55	0.135	10.94	5.092	1.090	0.009	0.008

**Table 2** shows that during the period of time from 4 to 6 years, Equation 9 was not met. However, at the ages of 8 and 10 years, the values of G'/(G−b0) were very close to the values of H'/H . The significant differences took place at the ages of 12 years due to the artificial thinning at the ages of 10 and 11 years. The similarity between the values of G'/(G−b0) and H'/H was quickly achieved at the age of 14 years and maintained until the stand was clear-cut at the age of 30 years.

### 3.3 Analysis of stand growth balance status

For real plantations, at the beginning, the growth of tree basal area (or D) and height is allometric, that is, at different growth rates, and Equation 9 is often not met, implying that the growth status of stands remains unbalanced. During this time period, generally, the values of $G'/\left(G-b_{0}\right)$ and H'/H are greater than those at the stage of the stand growth balance status. As trees grow, the stands develop towards the status of being fully stocked and eventually reach their balance status of growth. After that, the stands will maintain the balance status of growth for a certain number of years until self-thinning happens, which will break the balance status of growth. The number of years at which the stands stay at the growth balance status may vary depending on tree species and site condition. After self-thinning or artificial thinning, trees acquire their space needed for growing. The trees continue to grow until the stands recover back the balance status, that is, the maximum size-density line. The process can be theoretically accounted for as follows:

Based on Equation 9, when $b_{1}^{'}=0$ , $\frac{{\left(G-b_{0}\right)}^{'}}{G-b_{0}}=\frac{G^{\prime }}{G-b_{0}}=\frac{H^{\prime }}{H}$ . Because of a negative value of $b_{0}$ , $\frac{G^{\prime }}{G}>\frac{G^{\prime }}{G-b_{0}}$ . As G increases, ( $G-b_{0}$ ) → G, and then $\frac{G^{\prime }}{G-b_{0}}$ → $\frac{G^{\prime }}{G}$ and $\frac{G^{\prime }}{G-b_{0}}=\frac{H^{\prime }}{H}\;$ → $\frac{G^{\prime }}{G}=\frac{H^{\prime }}{H}$ . This indicates that for fully stocked stands, maintaining the balance status of growth requires the equally relative growth rates of height and basal area. If the number of trees is not changed, then there is $\frac{G^{\prime }}{G}=\frac{H^{\prime }}{H}\to \frac{g^{\prime }}{g}=\frac{H^{\prime }}{H}\to \frac{2D^{\prime }}{D}=\frac{H^{\prime }}{H}$ . This implies that maintaining the balance status of growth requires the growth rate of height be close to the doubled growth rate of D.

In **Table 2**, there was a big difference in the $\frac{G^{\prime }}{G-b_{0}}$ value from that of $\frac{H^{\prime }}{H}$ from the age of 4 to 6 years and their values became very similar to each other at the ages of 8 years and 10 years because the stand got fully stocked. The artificial thinning at the ages of 10 and 11 years led to a big difference in the $\frac{G^{\prime }}{G-b_{0}}$ value from that of $\frac{H^{\prime }}{H}$ at the age of 12 years. As the trees grew, the values of $\frac{G^{\prime }}{G-b_{0}}$ became similar to those of $\frac{H^{\prime }}{H}$ again. After the year of 22, the difference of the values could be almost ignored.

**Figure 3:**
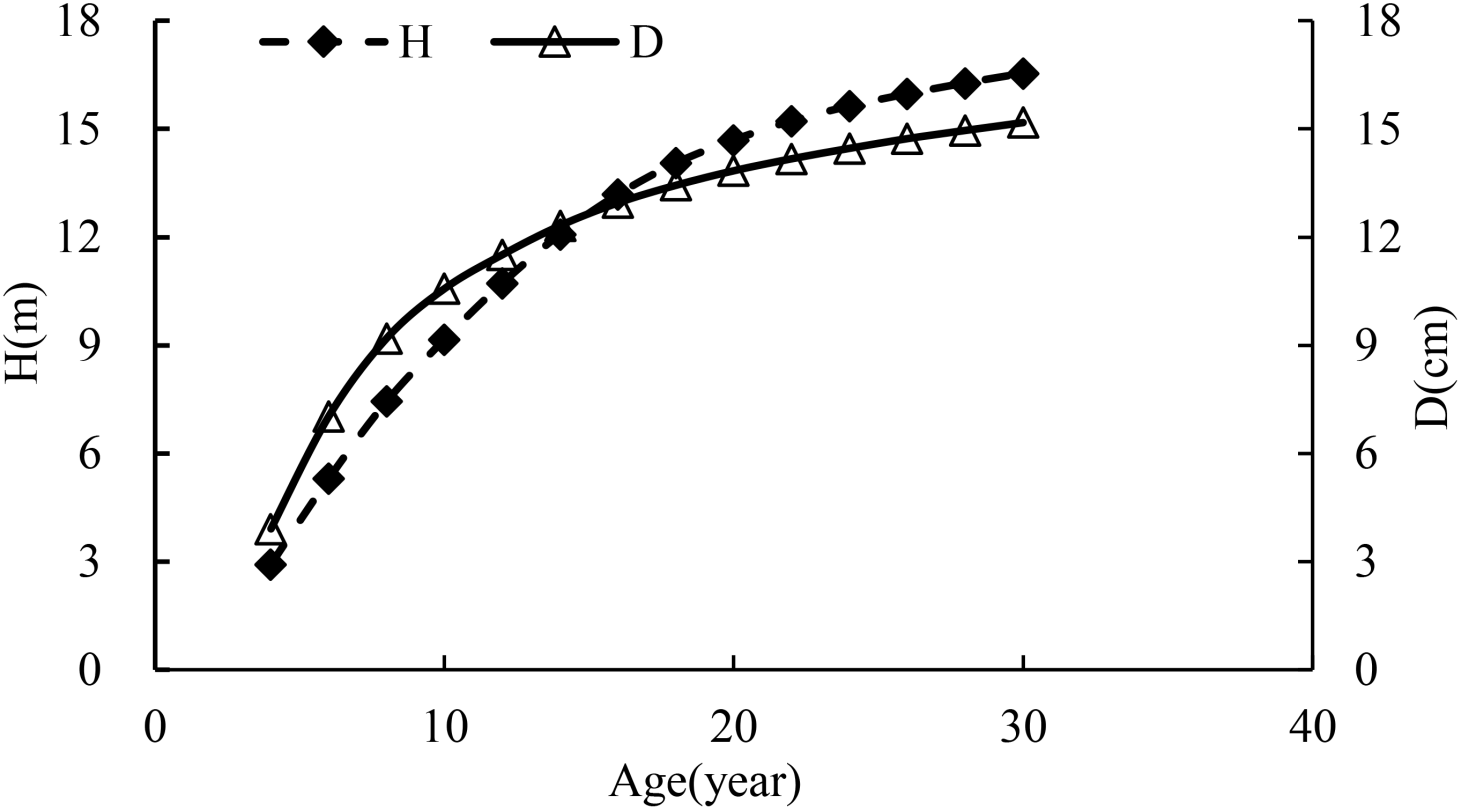
The growth curves of average height and *D* in the stand.

In **Table 2** and **Figure 3**, as the trees grew, the ratio of *H* to *D* increased with a great change occurring during the period of 10 to 12 years. During this time period, the growth rates of *D* and height were 4.1% and 7.3%, respectively, implying that the growth rate of height was smaller than the doubled growth rate of *D*. That is, $\frac{2D^{\prime }}{D}-\frac{H^{\prime }}{H}>0$ . At the stage of the stand growth balance status, theoretically, $\frac{2D^{\prime }}{D}\;$ should be close to $\frac{H^{\prime }}{H}$ . However, the management thinning broke the balance status. On the other hand, without the management thinning, the stand would grow for a certain time period but not very long, and then the different growth rates of the average diameter and height would break the growth balance status of the stand in which self-thinning would happen. In addition, the growth curves of height and *D* in **Table 2** and **Figure 3** also implied the variable stem form factors over time (**Table 1**) due to the different growth rates of height and *D*.

### 3.4 Stand density index and self-thinning equation

Based on the measurements of average height and *D* in the stand, line segment 4, and Equation 7, the following SDI was derived:


10 $$SDI=N{\left(\frac{D_{0}}{D}\right)}^{-2}$$

where the standard $D_{0}$ is 15 cm and b=−2 . Using Eq. (10) with b=−2 and b=−1.605 , respectively, the values of maximum SDI for different average heights and *D* are calculated in **Table 3**.

**Table 3:** The values of the maximum stand density index for different average heights and *D* (D_0_ = 15 cm).

*H*	*D*	*g*	*G*	*N*	*SDI* (b = −2)	*SDI* (b = −1.605)
10	10	0.0079	29.65	3,775	1,678	1,969
	12	0.0113	29.65	2,622	1,678	1,833
	14	0.0154	29.65	1,926	1,678	1,724
	16	0.0201	29.65	1,475	1,678	1,636
12	10	0.0079	37.68	4,797	2,132	2,502
	12	0.0113	37.68	3,331	2,132	2,329
	14	0.0154	37.68	2,448	2,132	2,191
	16	0.0201	37.68	1,874	2,132	2,078
14	12	0.0113	45.70	4,041	2,586	2,824
	14	0.0154	45.70	2,969	2,586	2,658
	16	0.0201	45.70	2,273	2,586	2,521
	18	0.0254	45.70	1,796	2,586	2,406
16	12	0.0113	53.73	4,750	3,040	3,320
	14	0.0154	53.73	3,490	3,040	3,124
	16	0.0201	53.73	2,672	3,040	2,964
	18	0.0254	53.73	2,111	3,040	2,829

In **Table 3**, it was found that when the stand maintained the maximum size-density, the SDI increased as the average height increased for both b=−2 and b=−1.605 , implying that the SDI varied. Given an average height, moreover, $N=A\cdot D^{-2}$ led to an invariable SDI value, while $N=A\cdot D^{-1.605}$ resulted in variable SDI values, implying that $N=A\cdot D^{-1.605}$ did not work, while $N=A\cdot D^{-2}$ worked well. This accounted for the reasonability of b=−2 with A = $\frac{4}{\pi }\left(b_{0}+b_{1}H\right)$ . The parameter A varied and its value is a function of average height and relative stand density, but not dependent on site condition and stand age.

Based on Equation 10, Equation 3 and line segment 4, the following stand maximum size-density index equation was obtained:


11 $$SDI=\frac{G_{1.0}}{g}\times {\left(\frac{D}{D_{0}}\right)}^{2}=\frac{4G_{1.0}}{\pi D^{2}}\times {\left(\frac{D}{D_{0}}\right)}^{2}=\frac{4}{\pi }G_{1.0}D_{0}^{-2}=\frac{4\left(4.0123H-10.471\right)}{\pi }\times D_{0}^{-2}=A\times D_{0}^{-2}$$

The results of SDI obtained using Eq. (11) were consistent with the corresponding values listed in **Table 3**. The stand density index (SDI) of the stand can be calculated using the following relationship, as shown in Equation 12


12 $$SDI=N\cdot {\left(\frac{D}{D_{0}}\right)}^{2}=\frac{G}{g}{\left(\frac{D}{D_{0}}\right)}^{2}=\frac{4G}{\pi D^{2}}{\left(\frac{D}{D_{0}}\right)}^{2}=\frac{4}{\pi }\cdot GD_{0}^{-2}$$

Based on the data in **Table 2**, the relationship between tree height H and diameter D can be expressed using Equation 13


13 $$H=0.056D^{2}+0.1758D+1.3007,\;R^{2}=0.998$$

Combining Equation 7 and $A=\frac{4\left(4.0123H-10.471\right)}{\pi }$ led to


14 $$N_{1.0}=\frac{4}{\pi }\times \left(4.0123H-10.471\right)\times D^{-2}$$

**Figure 4:**
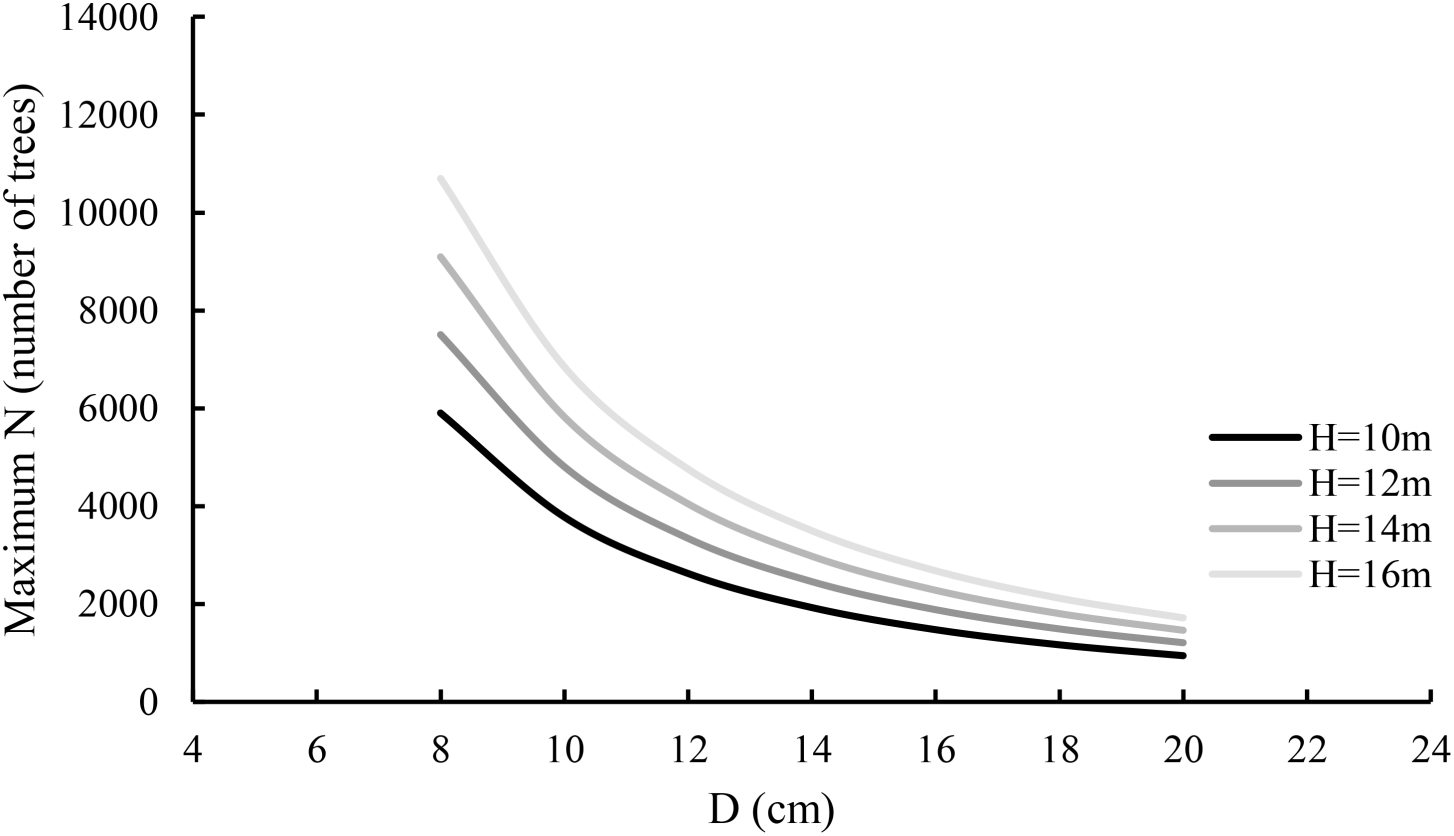
The relationship of maximum stand density N_1.0_ with average diameter and average height.

In Equations 11, 14, the estimates of intercept and slope related to A have the statistical test values of −12,108 and 34,652 with p-values close to zero and are significantly different from zero, implying that the average tree height has a significant contribution to *G*_1.0_ and thus to A, SDI, and *N*_1.0_. Equation 14 implies that the maximum size-density line varies depending on both average diameter and height. Equation 7 is generalized and Equation 14 is regarded as its specific case, only applied to the stand studied in this article and may not be applied to other planted stands or plantations. Based on Equation 14, the maximum size-density lines were graphed against average diameter and average height in **Figure 4**. Given a tree diameter, the taller the tree, the larger the maximum stand density. This is mainly because in a fully stocked stand, for the trees with the same diameter, the taller trees grow up for obtaining light and often have smaller tree crowns. In practice, this is widely noticed. On the other hand, given a tree height, the greater the diameter, the smaller the maximum stand density, which is also reasonable.

Similarly, Equation 8 is also generalized in which the average tree volume or biomass in a fully stocked stand is dependent on both the number of trees and average height. Based on the data in this study, the following relationship of the stand volume with average tree height was obtained:


15 $$V_{1.0}=7.1715H^{1.5041},\;R^{2}=0.9929$$

Combining Equation 8 and Equation 15 resulted in the following self-thinning equation for the Chinese fir stand, Equation 16, as a specific case of Equation 8:


16 $$v=7.1715H^{1.5041}N_{1.0}^{-1}$$

**Figure 5:**
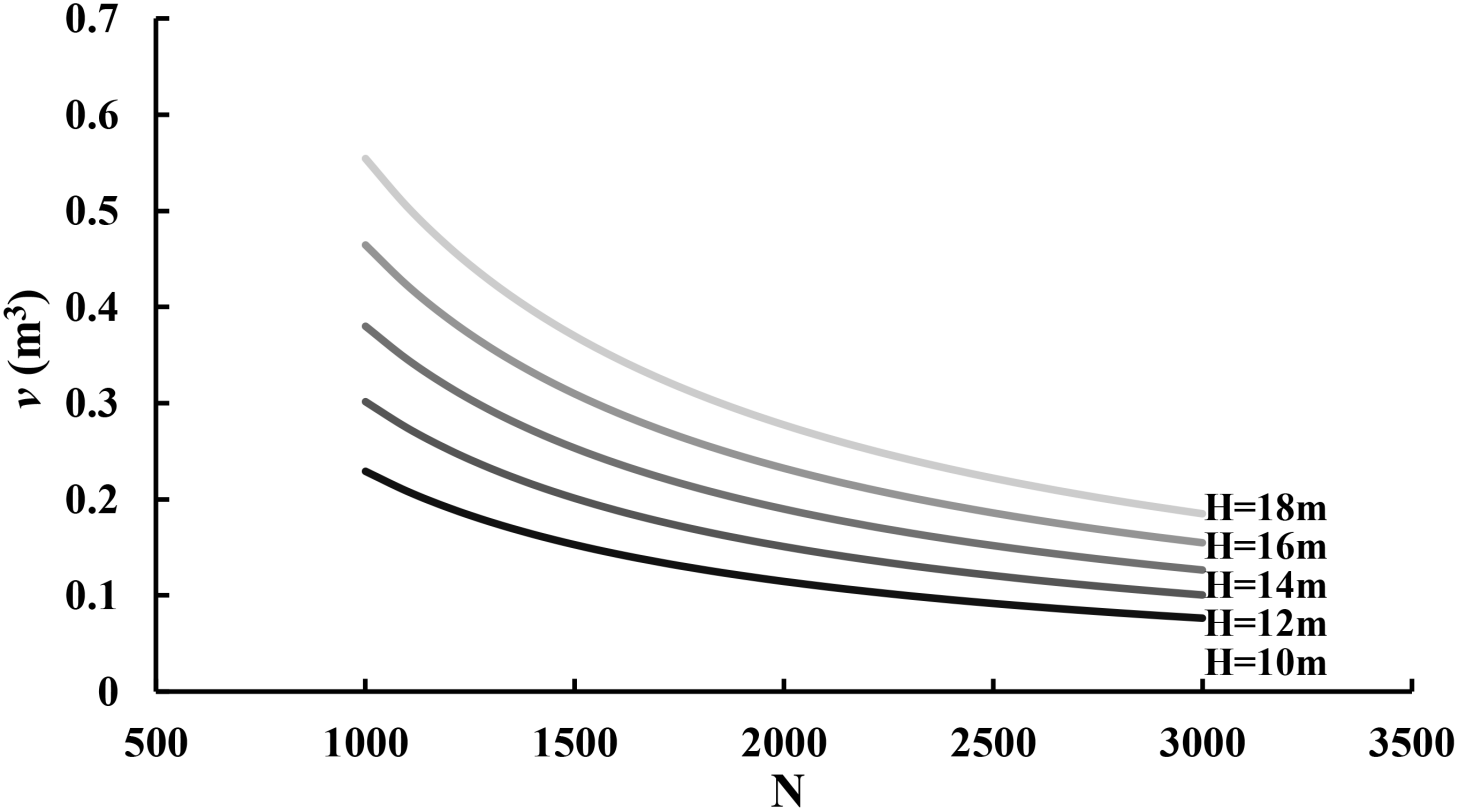
The relationship of tree volume (v) with the number of trees per hectare (N) and average tree height (H).

In Equations 15, 16, the estimates of the intercept and slope for log-transformation have statistical test values of 41.040 and 11.149 with p-values close to zero, implying that the estimates are statistically significantly different from zero and thus the average tree height significantly contributes to the stand and average tree volume. **Figure 5** demonstrated that the tree volume varied depending on both the number of trees per hectare and tree average height. Given a tree height, the tree volume decreases as the stand density increases. Given a stand density, the tree volume increases as the tree height increases. This implies that the self-thinning boundary line is a function of stand density and average height.

### 3.5 Application of *G/H*

Based on line segment 4, when a stand maintains the balance status of growth, *G*_1.0_ and *H* are related to each other: $G_{1.0}=b_{0}+b_{1}H$ . There is $\frac{G_{1.0}}{H}=\frac{b_{0}}{H}+b_{1}$ . Because $b_{0}$ is negative, as the average height increases, the absolute value of $\frac{b_{0}}{H}$ decreases, while $\frac{G_{1.0}}{H}$ increases and approaches to $b_{1}$ . The $G_{1.0}$ is the maximum total basal area of a fully stocked stand and $\frac{G_{1.0}}{H}$ thus implies that the average height determines the potential maximum productivity of a fully stocked stand. In a real stand, the ratio of the G/H to $G_{1.0}/H$ means the relative stand density, $G/G_{1.0}$ . Therefore, G/H can be regarded as an indirect indicator of relative stand density, simply called G/H ratio. This ratio is a function of stand average height and diameter and number of trees, but not site condition and stand age.

At the stage of the growth balance status with the invariable number of trees, there is $\frac{G_{1.0}}{H}=\frac{N\text{g}}{H}=\frac{a}{H}+b\to \frac{\text{g}}{H}=\frac{\frac{a}{H}+b}{N}$ . The ratio $\frac{\text{g}}{H}$ of the average tree basal area to the average height decreases as the number of trees increases. On the other hand, **Table 2** shows that the shorter the tree, the smaller the $\frac{\text{g}}{H}$ . These imply that at the stage of the growth balance status, increasing the number of trees will lead to shorter trees, which would potentially result in the earlier occurrence of self-thinning.

## 4 Discussion

Determining the maximum size-density line is critical for decision-making of management and planning, such as adaptive silviculture, thinning, and harvesting, for forests and especially plantations. The most important techniques include the selection of stands that are fully stocked and the development of the maximum size-density line. The traditional methods used to select the fully stocked stands often lead to uncertainty due to the lack of measures used to quantify the status of fully stocked stands (Bi et al., 2000; Newton, 2006; Osawa, 1995; Solomon and Zhang, 2002; Yoda et al., 1963).

In this study, the concept of growth balance status was proposed. At the stage of growth balance status, stands are fully stocked with an invariable slope of the relationship ( $G_{1.0}=b_{0}+b_{1}H$ ) between the stand basal area and average height and $\frac{G^{\prime }}{G-b_{0}}=\frac{H^{\prime }}{H}$ met, the growth rate of the per-unit basal area being similar to that of per-unit average height, and self-thinning has not happened. Artificial or self-thinning may break the growth balance status: $\;\frac{G^{\prime }}{G-b_{0}}=\frac{H^{\prime }}{H}$ , but the change of stand density due to thinning will adjust the growth of the basal area and height, which will make the stands eventually get back to the growth balance status. Thus, the growth balance status, $\frac{G^{\prime }}{G-b_{0}}=\frac{H^{\prime }}{H}$ , can provide a potential measure for the selection of fully stocked stands. In practice, the existing stands can be first visually interpreted for their status of being fully stocked, and the parameters (*D* and *H*) and their growth of the existing stands can be then measured and analyzed using $\frac{G^{\prime }}{G-b_{0}}=\frac{H^{\prime }}{H}$ to make a decision on whether or not the stands have been fully stocked and can be used to develop the maximum size-density line. For plantations, because of different site conditions and different numbers of trees planted, the time needed for stands to reach the growth balance status and the length of time for the stands maintaining the growth balance status may vary. However, the growth balance status does exist and can be used for the selection of fully stocked stands.

In this study, a generalized maximum size-density equation and a generalized SDI equation: $N_{1.0}=AD^{-2}$ and $SDI=AD_{0}^{2}$ were proposed based on the theoretical assumption and validation of the obtained data. The proposed equations are different from those of Reineke. In Reineke’s equations, A is a parameter varying only by tree species and the exponent of D is a universal constant, −1.605. In the proposed equations, *A* varies not only by tree species but also depending on average height, and the exponent of *D* is −2, not −1.605. For the Chinese fir plantation studied, A=4(4.0123H−10.471)/π is a specific case of the generalized equations. Moreover, a generalized self-thinning equation: $\text{v}=\text{k}H^{q}N_{1.0}^{-1}$ , or $\text{w}=c_{1}H^{q}N_{1.0}^{-1}$ , was also proposed in this study, which shows that the average tree volume or biomass is a function of both stand density and average height. As a specific case of the generalized equation, the self-thinning equation with k = 7.1715 and q = 1.5041 was obtained for the Chinese fir plantation studied. The proposed equation is different from the self-thinning equation of Yoda et al. (1963) in which the average tree volume or biomass is only a function of stand density with an exponent of −3/2.

In this study, the proposed generalized equations were first theoretically derived based on the assumption that tree diameter and height growth are allometric and determined by both tree crown and height (Weller, 1987a). This is because tree growth is achieved by biomass accumulation through absorption of materials (light, water and nutrients) and photosynthesis. During this process, the size of tree crown and tree height play a critical role. Tree diameter is highly correlated with tree crown (Fu et al., 2008). Moreover, tree height has a great effect on tree competition (Burkhart and Tomé, 2012; Long et al., 2020). The competition will result in different types of trees such as dominant, co-dominant, average, and suppressed trees. In addition, in a fully stocked stand, although the growth balance status exist, in which the basal area growth balances the height growth, the growth balance status will only last a short time. The allometric growth of diameter and height will break the growth balance status and lead to self-thinning. The results of this study also showed that the average tree height had statistically significant contributions in both maximum size-density and self-thinning equations. This implies that tree average height cannot be neglected in developing both the maximum size-density law and the self-thinning boundary line. This finding is supported by previous studies (i.e., Burkhart, 2013; Lynch et al., 2007; Vanclay and Sands, 2009). Both Reineke’s maximum size-density and Yoda’s self-thinning equation ignore the effect of tree height, which explains why many authors obtained different values of the exponent based on Reineke’s and Yoda’s equations (i.e., Rivoire and Moguedee, 2012; Pretzsch and Biber, 2005). The proposed equations are theoretically appropriate for other species plantations but may have different values of parameters A, k, and q due to variable relationships of basal area and volume or biomass with average tree height by different tree species. However, the appropriateness of the proposed equations to be applied to other forests needs further validation based on field measurements.

The analysis of the tree growth in the studied stand revealed that as the trees grew, the stand reached its maximum size-density and became fully stocked. During the process, the values of H/D (**Table 2**) changed, which implies that the ratio of the stand G/H also varied. Based on the relationship of G_1.0_ with H, $G_{1.0}=b_{0}+b_{1}H$ , for fully stocked stands; however, G_1.0_/H has a limited value, b_1_, the slope of the G_1.0_–H relationship. Given a site condition, whether or not the limited value differs from tree species to species needs to be further investigated.

This study was conducted based on the data from the same stand. Analyzing the growth process of the trees for 30 years revealed the characteristics of the stand parameters and the relationships among them. Moreover, in this study, all the trees in the stand were cut and their stem analyses were carried out through vertically sawing each of the stems in half and measuring their diameters at an interval of 2 m and their heights for every 2 years. The analysis greatly decreased the errors of the measurements and revealed the real growth processes of the trees. The result of this study showed that the slope of the proposed SDI equation was not related to site condition and stand age. The conclusions drawn in this study was reasonable and reliable. However, it had to be pointed out that the maximum size-density line in this study was determined based on line segment 4 fit using the time-series data from 4 to 10 years at which the stand just had the canopy closure. The determination of the tree crown cover might be associated with uncertainty. How the uncertainty due to measuring the canopy cover affects the maximum size-density line should be further explored in future studies.

In addition, in this study, the relationship of the stand average diameter and height with the stand density was explored. The growth of the upper stem diameter and the change of biomass density were neglected. In the fully stocked stands, in fact, owing to tree competition, the trees often grow up to obtain space for growing, which will increase the values of height timing stem form (**Table 1**). As trees grow, the change of biomass density is also important but was ignored in this study due to lack of observations. Generally, when stands become fully stocked, the increase of tree volume will be slowed down, but biomass density increases. There are also other factors that affect maximum size-density lines, including the adaptation of trees to the fully stocked environment, the effective use of light, and shading (Zeide, 2005). All the relevant aspects related to growth of trees should be studied in the future for optimization of maximum size-density equations and stand density management.

## 5 Conclusions

Based on the theoretical assumption that growth of stand parameters is allometric and mainly determined by tree crown and height, and the validation from the time-series data of measurements from a 30-year Chinese fir plantation, this study led to the following novel conclusions:

(1) The growth balance status of fully stocked stands was proposed, in which before self-thinning, the growth rate of per-unit stand basal area is similar to that of per-unit average height, that is, $\frac{G^{\prime }}{G-b_{0}}=\frac{H^{\prime }}{H}$ is met and approaches to a constant, b_1_, the slope of the stand basal area–average height relationship. The growth balance status can be applied to the selection of fully stocked stands. However, the growth balance status of fully stocked stands may not last a long time and will be broken by the allometric growth of diameter and height, which will lead to self-thinning. This implies that the assumption of the same diameter and height growth rates in the theory of Reineke’s maximum size-density law was not supported by the result of this study.

(2) In this study, a limited value of G_1.0_/H was found. This implies that when the average height stops growing, the stand total basal area will no longer increase. At this stage, the stand volume or biomass becomes constant because the amount of tree growth will be cancelled out by the amount of loss due to self-thinning. This conclusion is consistent with the finding of Fang (1992).

(3) In this study, the generalized maximum size-density and SDI equations were proposed: $N_{1.0}=AD^{-2}$ and $\text{SDI}=\text{A}D_{0}^{2}$ with $G_{1.0}=b_{0}+b_{1}H$ and $A=4\left(b_{0}+b_{1}H\right)/\pi$ . The exponent of the maximum size-density law is −2. Both the maximum size-density and SDI vary dependent on the average tree height in addition to D. The average tree height significantly contributes to N_1.0_ and SDI. The conclusion differs from the widely used maximum size-density law of Reineke (1933).

(4) This study also resulted in a generalized self-thinning equation: $\text{v}=\text{k}H^{q}N_{1.0}^{-1}$ , or $\text{w}=c_{1}H^{q}N_{1.0}^{-1}$ . The average tree height has a significant contribution to the average tree volume or biomass. This implies that in a fully stocked stand, the tree volume or biomass varies depending not only on the number of trees but also on average tree height. Thus, the self-thinning rule proposed by Yoda et al. (1963): $\text{w}=\text{k}N^{-\frac{3}{2}}$ , is not supported by this study.

It is expected that the findings can help to enhance our understanding of the maximum size-density law and the self-thinning rule and provide useful tools for stand density management of Chinese fir plantations.

## Data Availability

The original contributions presented in the study are included in the article/supplementary material. Further inquiries can be directed to the corresponding author.
